# Cutaneous drug-reactions to nevirapine: study of risk factors in 268 HIV-infected patients

**DOI:** 10.1186/1471-2334-14-S2-P49

**Published:** 2014-05-23

**Authors:** L Badaoui, G Dabo, H Lamdini, R Bensghir, A Oulad Lahsen, M Sodqi, L Marih, A Chakib, K Marhoum El Filali

**Affiliations:** 1 Ibn Rochd University Hospital, Casablanca, Morocco

## Introduction

Several studies have shown a high prevalence of rash induced by nevirapine. The aim of this study was to identify risk factors associated with the occurrence of rash during the treatment with nevirapine of HIV-infected patients.

## Materials and methods

A retrospective study was conducted in the infectious department diseases between January 2007 and October 2013. The study included all HIV-infected patients receiving HAART regimens that included nevirapine. The following data were collected: age, sex, CDC classification of HIV, CD4 and lymphocyte counts, plasma HIV RNA load, history of drug allergy, concomitant medication.

## Results

During the study period, 268 HIV-infected patients were treated with HAART regimens including nevirapine. Nineteen developed cutaneous drug-reactions attributable to nevirapine (7. 09%). 16 women and 3 men; mean age: 38 (± 12) years old. The mean initial CD4 was 320 cell/mm3, we observed 17 cases of maculopapular exanthema and 2 case of DRESS, the hepatic cytolysis retouved in 5 cases. Female gender (84%), plasma HIV RNA load > 10 000 copies/ml (100%) constituted risk factors associated with rash. The niverapine has switched either Efavirenz or IP; no allergic event was detected after changing the niverapine.

## Conclusion

The number of HIV-infected patients who are newly exposed to nevirapine is increasing worldwide. To minimize toxicity, clinicians must adhere to dosing guidelines, avoid prescribing the drug in patients with known increased risk of toxicity, and promptly recognize toxicities, which are mainly cutaneous and hepatic.

